# Nanomedicine drug delivery in South Africa: a retrospective study on research, funding and collaboration

**DOI:** 10.3389/fphar.2023.1317137

**Published:** 2024-01-03

**Authors:** Faatiema Salie, Trust Saidi

**Affiliations:** ^1^ Division of Biomedical Engineering, Department of Human Biology, University of Cape Town, Cape Town, South Africa; ^2^ Centre for Technology, Innovation and Culture, University of Oslo, Oslo, Norway; ^3^ Athena Institute, Faculty of Science, Vrije Universiteit Amsterdam, Amsterdam, Netherlands

**Keywords:** nanomedicine, drug delivery, disease, funding, actor-collaboration networks, networks, nanotechnology

## Abstract

After nearly two decades of substantial investment in the field of nanomedicine within South Africa, this study undertakes an investigation into the specific diseases that have been targeted for research and development, as well as the key actors and collaborative networks involved in this burgeoning field. To accomplish this, the study adopts a mixed-method approach, combining bibliometric and scientometric techniques alongside a comprehensive review of existing literature. The study’s findings illuminate that the diseases selected for emphasis in nanomedicine research closely align with the prevalent health challenges faced by South Africa. Notably, these ailments encompass cancer, bacterial infections, and tuberculosis, all of which significantly contribute to the country’s disease burden. Furthermore, the investigation highlights that research-intensive South African universities play a pivotal role as the primary actors in advancing nanomedicine initiatives. Over time, collaborative endeavors among these key actors have seen a noteworthy upswing. These collaborations have fostered robust connections between South African institutions and counterparts in Asian nations and the Middle East. It is worth emphasizing that nanomedicine is a resource-intensive field, necessitating substantial capital investment. Collaborative initiatives have, in turn, granted access to critical infrastructure and materials that would have otherwise been beyond the reach of some participating entities. Remarkably, these collaborative partnerships have not only facilitated scientific progress but have also cultivated social capital and trust among involved stakeholders. These valuable intangible assets hold great potential as South Africa advances towards more exploitative phases of technology development within the domain of nanomedicine. Moreover, South Africa is strategically positioning itself to cultivate a critical mass of expertise in nanomedicine, recognising the significance of skilled human resources in harnessing the full potential of this technology in the future.

**Systematic Review Registration:** https://www.ncbi.nlm.nih.gov/pmc/articles/PMC6173875/

## Introduction

Nanomedicine is one of the most exciting applications of nanotechnology for the diagnosis and therapy of a variety of diseases as well as in regenerative medicine (Boulaiz et al., 2011; Chang et al., 2015; de Oliveira et al., 2016). This emerging and evolving field holds the potential of providing significant breakthroughs in developing countries in terms of improved and cost-effective healthcare, which is a crucial factor in making medicines and treatments available and affordable (Chang et al., 2015). It is envisaged that the continual development of nanomedicines will offer benefits such as improved efficacy, bioavailability, dose–response, targeting ability and safety compared to conventional medicines (Bharali and Mousa, 2010; Ventola, 2012). Given the broad applications of nanomedicine, questions are emerging on issues of equity, development and access to nanomedicine, particularly in developing countries (Cozzens and Wetmore, 2010). There are concerns that limiting access to nanomedicine could create a ‘nano-divide’ between wealthy and poor countries, thereby exacerbating existing differences in health outcomes (Maclurcan, 2012). This may result in nanomedicine coming under the control of powerful interests and market forces and not towards the needs of the poor (Salamanca-Buentello and Daar, 2021).

In an effort to coordinate the exploitation of the technology, several developing countries have initiated and enacted nanotechnology plans for their respective countries (Salamanca-Buentello, Persad, Martin, Daar and Singer, 2005). South Africa is one such country as it launched its National Nanotechnology Strategy in 2005 with the main objectives of supporting long-term nanoscience research and exploration in the areas of health, among others (Molapisi, 2012). The country is emerging as one of the leaders in nanomedicine research and product development on the African continent (Mufamadi, 2019). In Africa, South Africa stands out as a country with better infrastructure for healthcare services and dedicated biomedical research, hence it is a significant player in nanomedicine research and product development on the continent (Dube and Ebrahim, 2017). The South African government has mobilized resources and investment towards the development of a critical mass of infrastructure, equipment, and human capital for the exploitation of nanotechnology (Dube and Ebrahim, 2017) and there is a need to assess the progress that the country has made so far. Approaching nanotechnology from its broad context encompassing fields as diverse as energy storage, water purification and molecular engineering can be overwhelming, and it poses the risk of missing the finer details that emerge from an in-depth analysis. The focus on nanomedicine is informed by the fact that South Africa is one of the countries facing the challenge of a high burden of disease such as HIV/AIDS and tuberculosis (TB) of which there are existing drugs for treatment (Narsai, Leufkens and Mantel-Teeuwisse, 2021). However, the challenge is on the effectiveness of the medicines due to poor solubility and bioavailability, which puts a demand for targeted drug delivery systems (Fasinu, Pillay, Ndesendo, du Toit and Choonara, 2011). Engineered nanoparticles and structures can be used as drug carriers for targeted delivery as they are capable of conveying therapeutically active molecules to the site of action, without having an effect on other organs and tissues (Ruggiero, Pastorino and Herrera, 2010; Mishra, Kesharwani, Amin and Iyer, 2017; Baranyai et al., 2021). This brings with it the advantage of facilitating relatively lower doses of drugs and increasing their therapeutic indices and safety profiles. An example of this important feature of nano delivery systems are orally administered nanomedicines that can overcome chemical and physical barriers in the human gut, such as the stomach pH, intestinal mucosal lining and selectively permeable membranes of enterocytes (Rudrapel et al., 2022). It is against this background that in this paper, we focus on the application of nanotechnology in medicine.

The paper is motivated by the systematic review protocol on nanomedicine for drug delivery in South Africa (Saidi, Fortuin and Douglas, 2018) and a foresight perspective of nanotechnology in South Africa based on a 20-year period scientometric analysis of the country’s nanotechnology publications (Masara, van der Poll and Maaza, 2021). While the latter focuses on a foresight perspective, this paper differs in that it approaches the study of nanomedicine in hindsight. In addition, this paper by focusing on nanomedicine in particular differs from that of Masara et al. (2021) which approaches nanotechnology in general and makes a recommendation for further studies to establish a system for classifying nanotechnology papers into economic sectors such as nanomedicine, nanoenergy, nanoagriculture and nanoelectronics. Taking a cue from that, this paper is anchored on three complementary objectives on the development of nanomedicine in South Africa. First the paper reviews the types of diseases that are targeted for nanomedicine drug delivery; second, it identifies the geographical settings of the authors of the publications and establishes the network for collaboration; and lastly it draws policy insights on how the country can exploit the opportunities and address challenges posed by nanomedicine. This is largely achieved through a combination of bibliometrics and scientometric techniques, including social network and keyword network analysis, and a review of government and actor institutions.

## Methodology

In this study, we make reference to the systematic review protocol on nanomedicine for drug delivery in South Africa (Saidi et al., 2018), to capture scientific knowledge that has been produced in this research area. We defined nanomedicine as the application of nanotechnology to the discipline of medicine which involves the use of nanoscale materials for the diagnosis, monitoring, control, prevention, and treatment of disease (Moghimi, Hunter and Murray, 2005; Tinkle et al., 2014). We searched databases, namely, PubMed, Scopus, and Web of Science, to identify scientific publications developed by South African organisations in nanomedicine drug delivery. The search phrases used in these databases have been included in Appendix 1.

**FIGURE 1:**
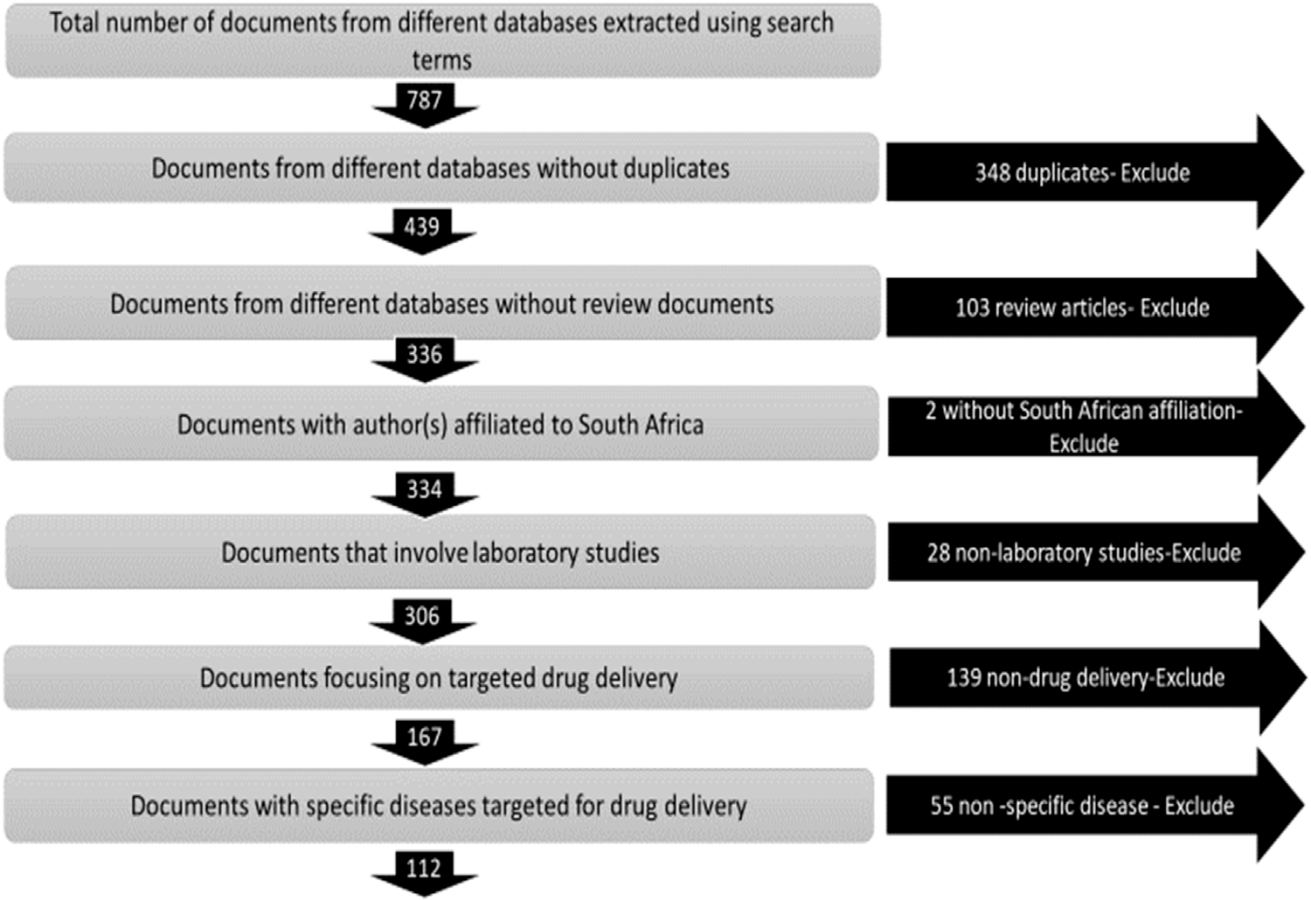
Inclusion and exclusion criteria for selection of scientific publications.

From the three databases, a total of 787 documents were extracted. The selection criteria, shown in Figure 1, was used as the basis for the inclusion and exclusion of documents.

The final dataset comprised 112 scientific publications. Bibliographic data was collected from these publications and serve as the primary dataset for determining the types of diseases that are targeted in nanomedicine drug delivery, identifying the organisations, their geographical settings and networks for collaboration, and funding mechanisms for work in nanomedicine drug delivery in South Africa.

### Actor-collaboration networks

We used co-authorship of scientific publications as a proxy for collaboration and perform social network analysis to illustrate the nature of relationships of actors who publish in nanomedicine drug delivery in South Africa. Author affiliation, at the organisational level, was extracted and recorded from each publication. Actor-collaboration networks were generated using UCINet (Borgatti, Everett and Freeman, 2002) and NetDraw (Borgatti, 2002). NetDraw’s spring embedded algorithm was used to draw the networks and nodes were manually manipulated to best illustrate network dynamics and relationships. In the actor-collaboration network, the organizational affiliations are the nodes (actors) and each instance of co-authorship is represented as an edge between nodes. The thickness of the edge is weighted according to the number of co-authored publications. Edges are undirected as co-authorship is considered as a reciprocal relationship. The nodes and edges combine to form components. Two nodes are part of the same component if there is a path connecting them.

Each node was assigned attributes based on sector and geographical location. All South African organisations were attributed ‘national’ and all other organisations were attributed ‘international’ however, in the networks the nodes are coloured by their geographic region. Each node was assigned to one of the following four sectors.

(1) Healthcare, which includes all hospital, including academic hospitals, clinics, and specialized healthcare facilities.
(2) University, which includes all forms of higher education organisations, such as universities, colleges, etc.
(3) Research institutions, which includes science councils, and research organisations other than universities.
(4) Industry, which includes individuals and organisations whose aim is to take products to market, usually to make a profit.

Network metrics of interest here are mainly for illustrative purposes. They include degree centrality and betweenness centrality. The degree centrality is a measure of the number of collaborations in which the node is involved serving as an indicator of how active the node is. It is calculated as the number of ties between a given node and other nodes in the network. Betweenness centrality is a measure of how often a node lies on the shortest path between two other nodes. Nodes with high betweenness centrality are considered to influence the flow of information across the network.

### Identifying disease through keyword networks

We used keyword networks to identify the research focus of nanomedicine drug delivery and its applications, as well as the interrelationship between research fields. Using the same dataset for the actor-collaboration networks, keyword networks were generated from keywords listed on the publication. Each keyword is represented as a node in the network, and an edge between two nodes indicates that those nodes appear on the same publication. Metrics of interest are again degree centrality and betweenness centrality, however, they take on a different interpretation in the keyword networks, i.e., degree centrality is a measure of how many research areas are connected to a given research area, while betweenness centrality is a measure of which research areas connects sub-sets of research areas. The popularity of a keyword (Choi et al., 2011) was also investigated, where popularity is based on how often they appear on articles.

### Funding instruments

In each publication, the Funding, Acknowledgements and Declaration of Interest sections were investigated to illicit who financed the project, and what infrastructure was accessed from collaborators, or others, in the project.

## Results

### Types of diseases

**FIGURE 2:**
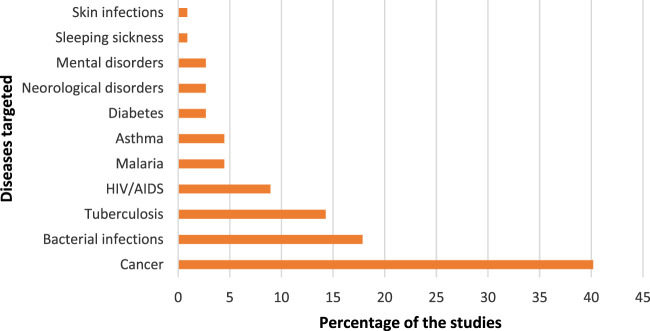
Targeted diseases of nanomedicine drug delivery in the manual assessment of scientific publications.

**FIGURE 3:**
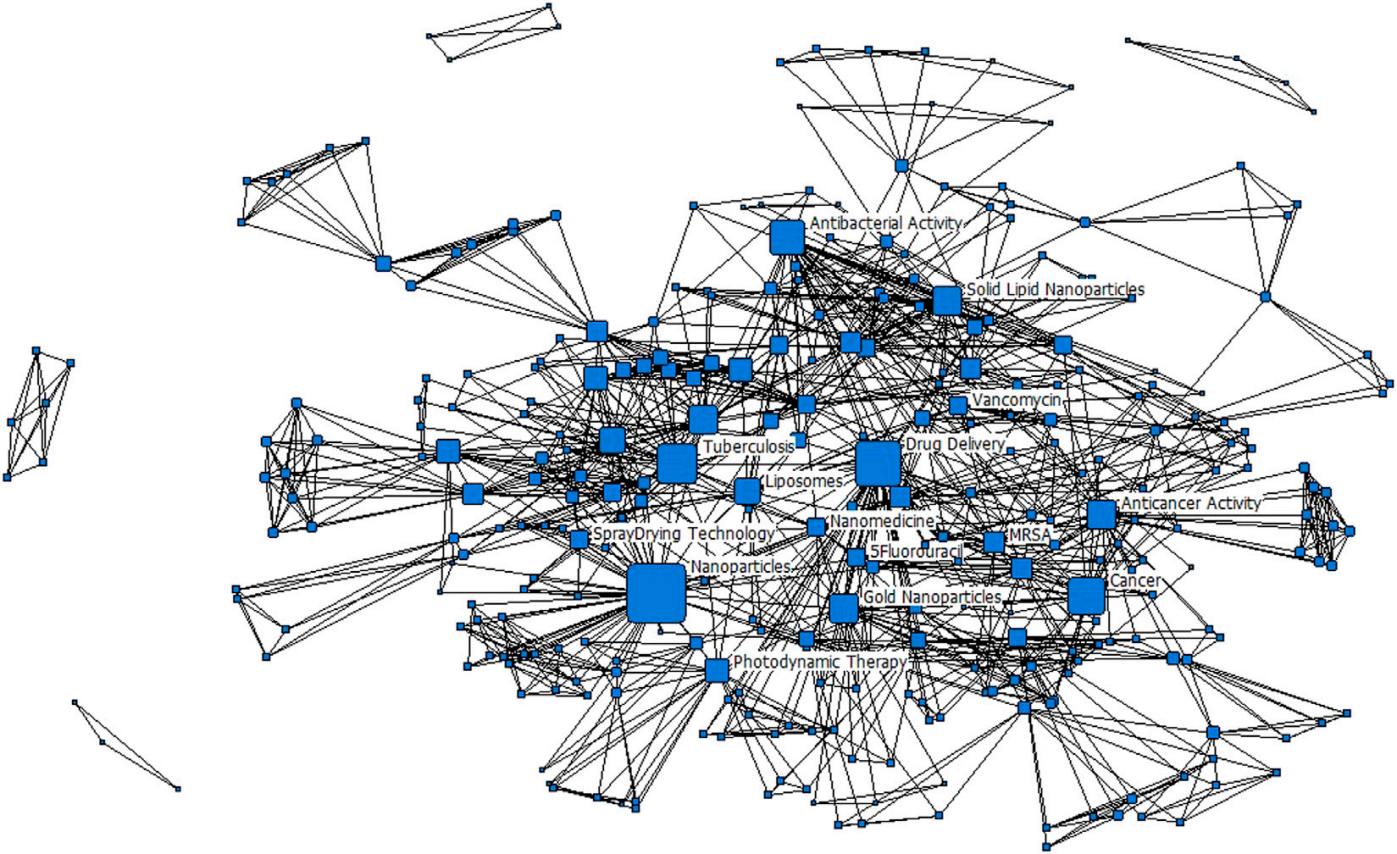
Keyword network for the period 2006–2020. Only Popular keywords have been labelled. Nodes are scaled to the degree centrality metric; edges are unweighted.

**FIGURE 4:**
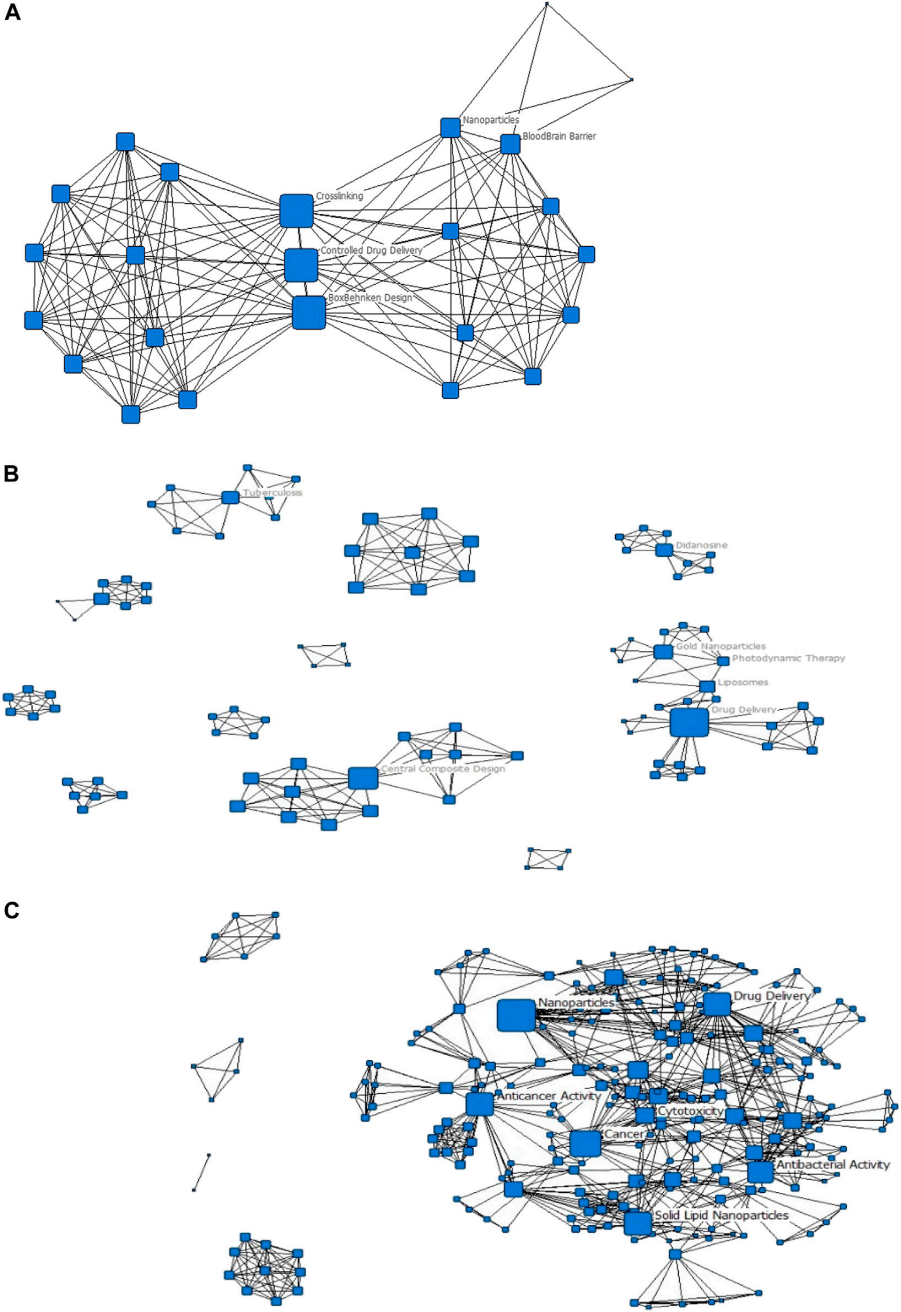
Evolution of the keyword networks **(A)** 2006–2010; **(B)** 2011–2015; **(C)** 2016–2020. All nodes have been scaled to the Degree centrality metric; only the highest-ranked nodes by degree and betweenness centrality have been labelled.

Ninety-one of the 112 (81%) journal articles had author-assigned keywords listed on them. To maintain homogeneity of the dataset, we did not assign keywords to the rest, but rather consider the 81% of articles to be reflective of the research activity of the actors in the network. Concurrently, we manually assessed the content of all the documents. The manual assessment captures that information that the keyword networks may have missed. In our manual assessment we developed Figure 2, while in our keyword networks we developed Table 1 and Figures 3, 4. As authors defined similar concepts differently, keywords were standardised. For example, the term ‘antibacterial activity’ encompasses ‘antibacterial’ and ‘antibacterial activity’ and ‘enhanced antibacterial activity’. After standardizing, 324 keywords were retained for further analysis, and the popularity of a given keyword was determined. Most keywords only appeared on one publication; 41 keywords appeared on two or more publications, and 15 keywords appeared on three or more publications. The list of the 15 most popular keywords is presented in Table 1.

**TABLE 1:** Popular keywords.

Keywords	Number of publications
Nanoparticles	13
Drug delivery	12
Antibacterial activity	9
Gold nanoparticles	9
Cancer	8
Photodynamic therapy	8
Tuberculosis	8
Solid lipid nanoparticles	7
Liposomes	6
Vancomycin	6
5-Fluorouracil	5
Anticancer activity	5
MRSA	5
Nanomedicine	5
Spray-drying technology	5

Unsurprisingly, the most popular keywords in the dataset are ‘Nanoparticles’ and ‘Drug delivery’, which really captures what the study set is about. From Figure 2, we see that nanomedicine is being applied for targeted drug delivery of several diseases of which cancer is the most common (40% of the 112 studies). ‘Cancer’ and ‘Photodynamic therapy’ appeared as a keyword on eight publications, while ‘Anticancer activity’ appeared on five publications. This came against a background in which cancer mortality rates were increasing in developing countries and South Africa was not an exception. Cancer is regarded as the second highest cause of death in South Africa and kills more people than other non-communicable diseases such hypertension and diabetes (Stefan, 2015). In 2014, cancer accounted for eight percent of the total deaths in South Africa (Made et al., 2017). The prevalence in the application of nanomedicine for cancer treatment is a global trend in which the technology is extensively employed. Nanomedicine has the potential for improving anticancer therapy by being able to improve the balance between efficacy and toxicity through the modulation of biodistribution and target site accumulation of systemically administered chemotherapeutic drugs (van der Meel et al., 2019). ‘Cytotoxicity’ is again, a popular keyword. Among the applications of nanomedicine for various types of cancer treatment, breast cancer is the most dominant. This could be due to breast cancer being the most commonly diagnosed cancer among South African women (Lince-Deroche et al., 2017).

The application of nanomedicine for bacterial infections constituted 18% of the studies, and the keyword ‘Antibacterial activity’ appeared on 12 of the publications. Bacterial infections could be broadly conceived, however, the common bacterial infections in South Africa include *staphylococcus aureus*, *serratia marcescens*, *klebsiella pneumoniae* and *salmonella* (Fraser, Mwatondo, Alimi, Varma and Vilas, 2020). The keywords show that methicillin resistant *staphylococcus aureus* (‘MRSA’) is a popular topic for targeted drug delivery in this dataset. The infectious ailments due to bacteria pose burden on public health and are major causes of morbidity and mortality in developing countries. For example, South Africa is regarded as the primary site of pneumococcal penicillin resistance surveillance with one of the highest reported rates in the world (Crowther-Gibson et al., 2011). The application of nanomedicine, particularly the use of nanoparticles has been highlighted as a promising solution to the challenges posed by existing antimicrobials (Masri, Anwar, Khan and Siddiqui, 2019). From the articles that were selected for this study, nanoparticles in the form of dendrimers, liposomes, metallic nanoparticles, and polymeric nanoparticles were utilized as delivery vehicles.

Tuberculosis (TB) contributed 14% of studies. The treatment of TB is a challenge and has resulted in the outbreak of multi-resistance drug TB (Saidi, Salie and Douglas, 2017). Nanomedicine is being deployed in already existing TB medicines to enhance their efficacy and minimise side effects such as toxicity. The history of TB in South Africa is characterised by a rise in multi-drug resistant strains which is further compounded by a large number of people living with HIV/AIDS, and who constitute the largest population on treatment for HIV in the world (Marsh et al., 2019). The HIV-prevalence in South Africa could be the reason for the 9% of the studies in the dataset. Although malaria is not prevalent in South Africa, the disease constituted 4% of the papers. The country is working towards eliminating the disease but the challenge is with high levels of imported asymptomatic malaria from neighboring countries (Raman et al., 2020).

The keyword network presented in Figure 3 shows a cohesive primary component, comprising all the popular keywords. There are four other components in the network which are established from single journal articles. The research focus of nanomedicine drug delivery in South Africa can therefore be considered to be quite well connected by some strong research focus areas. The network presented in Figure 3 is a summation for the entire period 2006 to 2020. In Figures 4A–C, the evolution of the keyword networks is presented.

The evolution networks show that the research focus of the South African nanomedicine drug delivery space has changed significantly over time. In the earliest recordings, Figure 4A, the research focus is on controlled drug delivery with applications addressing the blood-brain barrier. In the second timeframe, Figure 4B, we see that the research focus areas are very disconnected; this is illustrated by the 11 components in the network. Additionally, the popular keywords lie across four different components. This suggests that the research focus areas, of TB and drug delivery and photodynamic therapy and gold nanoparticles, for instance, stem from different initial research focus areas. The last timeframe, Figure 4C, shows a significantly more cohesive network (as with Figure 3), showing that over time, research focus areas have become aligned, or have centralized towards common focus areas, largely in cancer and antibacterial activity applications.

### Actor-collaboration networks

**FIGURE 5:**
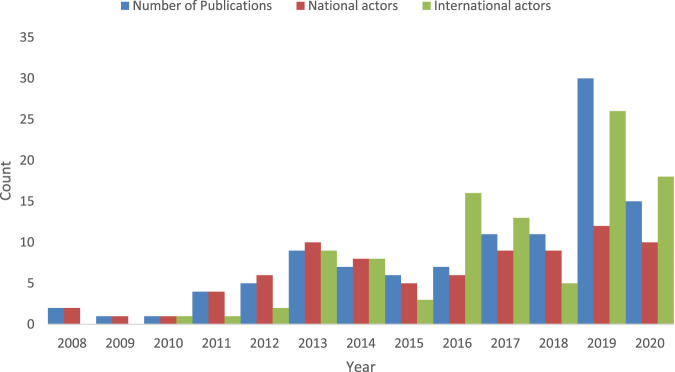
The number of publications, national and international actors over time. There were no publications produced in 2006 and 2007.

In Figure 5 we present how the number of publications, and numbers of national and international actors who contribute towards scientific knowledge development in nanomedicine drug delivery in South Africa changes over time. Before 2011 there are very few actors who produce publications, and no international presence. Over time, the number of national and international actors increase, and so too do the number of publications, peaking in 2019.

**FIGURE 6:**
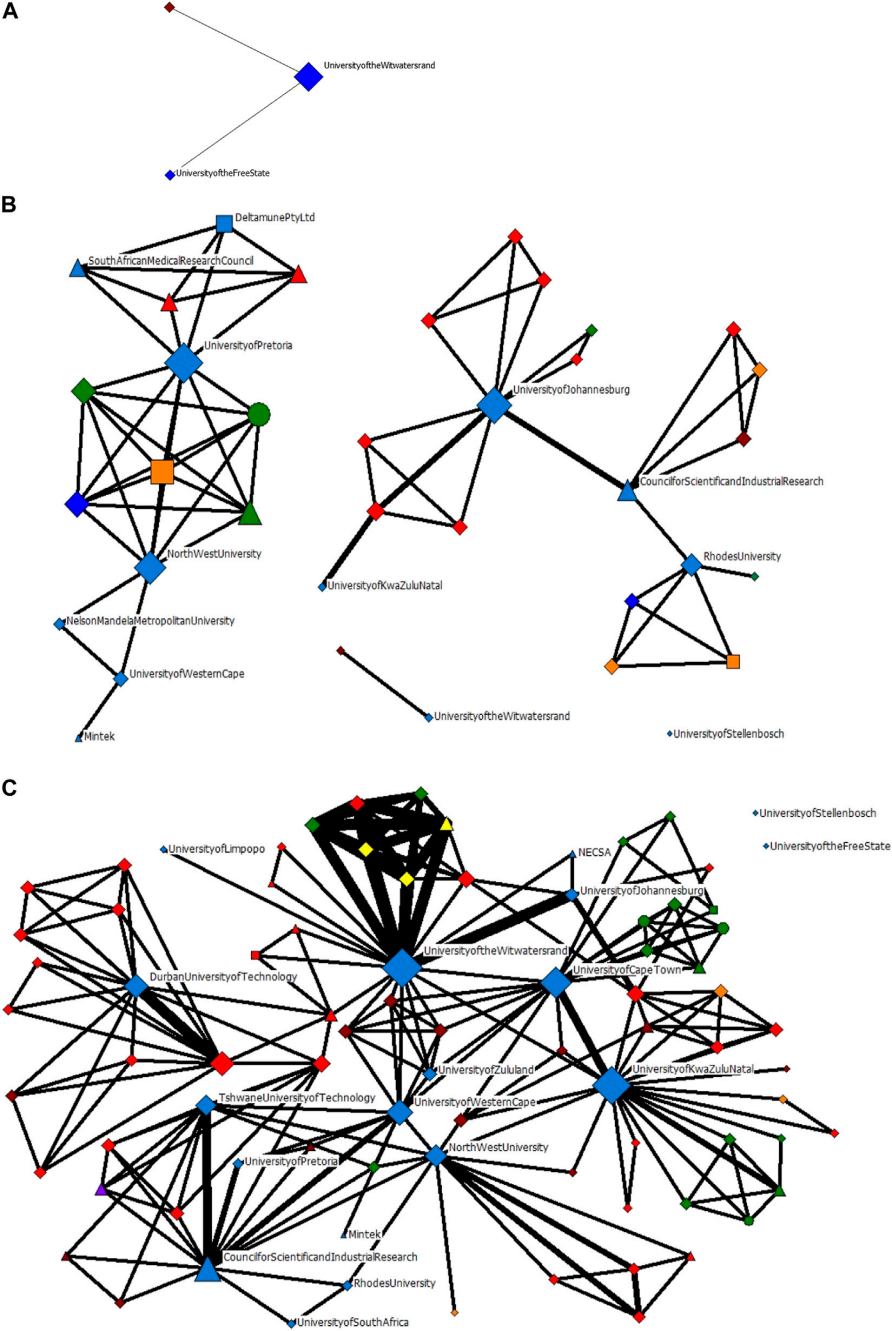
The evolution of actor-collaboration networks in nanomedicine for drug delivery in South Africa for the period **(A)** 2006–2010; **(B)** 2011–2015; and **(C)** 2011–2016.

The evolution of actor-collaboration networks are presented in Figure 6. The nodes have been coloured according to geographic region as Blue–South African; Maroon–Africa; Green–Europe; Red–Asia and the Middle East; Yellow–Australasia; Orange–North America; and Purple–South America. To maintain legibility of the network, only South African organisations have been labelled. The geographic and sectoral breakdown of the actors in the evolution network are presented in Table 2, while Table 3 further breaks the actors down at regional geographic levels. Universities and Research Institutions are by far the largest contributors to the scientific knowledge production. This may be biased towards scientific publications being a better indicator of knowledge production from these sectors. No South African Healthcare actors and only one South African Industry actor is present in this network; this may reflect the nascent innovation system, where healthcare and industry actors are not yet involved, and may only present in the technology development, clinical trials, and commercialization of the technology.

**TABLE 2:** Geographical and sectoral breakdown of actors who produce scientific publications in nanomedicine for drug delivery.

Network	University		Healthcare		Research Institutions		Industry		Total
	National	International	National	International	National	International	National	International	
2006–2010	2	1	0	0	0	0	0	0	3
2011–2015	9	17	0	1	3	3	1	2	36
2016–2020	15	49	0	3	3	11	0	2	83

**TABLE 3:** Regional breakdown of actors who produce scientific publications in nanomedicine for drug delivery.

	2006–2010	2011–2015	2016–2020
South Africa	2	13	18
Africa	0	2	12
Europe	0	5	16
Asia and Middle East	1	10	30
Australasia	0	0	3
North America	0	4	3
South America	0	2	1

The University of the Witwatersrand (WITS), the Council for Scientific and Industrial Research (CSIR), South African Medical Research Council (SAMRC), University of Pretoria (UP), North-West University (NWU), University of Johannesburg (UJ), University of KwaZulu Natal (UKZN), the University of Western Cape (UWC) and Rhodes University (RU) are all South African organisations who contribute immensely to the network over time.

The network characteristics of these national actors evolve: WITS, in the first two timeframes, has very few ties to the other network actors, but by the third timeframe, has both the highest degree and betweenness centrality, meaning that it is connected to the greatest number of nodes, as well as being the most well-established node for the flow of information through the network. Similarly, with UKZN, in the second timeframe it is only connected to one other node in the network, but by the third timeframe it has the second highest degree and betweenness centrality. Both these organisations illustrate how, in a short period of time, they establish a formidable network presence.

Initially, the networks are made up of mostly South African organisations. However, over time, there is a very large international presence, also from University and Research Institution sectors. After South African actors, actors from Asia and the Middle East are the second most prominent actors, and thirdly, European actors. The key actors, identified through the degree centrality and betweenness centrality metrics, are presented in Table 4, 5, respectively. Apart from the South African organisations, the other key actors by degree centrality are mainly formed from collaborations with Spanish and Indian organisations in the second time frame, and Indian and Australian organisations in the third time frame. Unsurprisingly, the highest-ranked actors by betweenness centrality are largely South African universities, who connect sub-groups of actors.

**TABLE 4:** Highest-ranked actors by degree centrality.

2006–2010		2011–2015		2016–2020	
Organisation	Value	Organisation	Value	Organisation	Value
University of Witwatersrand	4	University of Johannesburg	11	University of the Witwatersrand	41
Integral University	1	North-West University	8	University of KwaZulu Natal	24
University of the Free State	1	Council for Scientific and Industrial Research	6	International Medical University	23
		Indian Institute of Science	6	Centenary Institute	23
		University of Pretoria	6	University of Technology Sydney	23
		Institute of Bioengineering of Catalonia	6	Suresh Giyan Vihar University	23
		Hospital Clinic Universitat de Barcelona	6	University of Newcastle	23
		University of Barcelona	6	University of Cape Town	19
		Genzyme Corporation	6	Council for Scientific and Industrial Research	18
		University of Puerto Rico	6		

**TABLE 5:** Highest ranked actors by Betweenness centrality.

2006–2010		2011–2015		2016–2020	
Organisation	Value	Organisation	Value	Organisation	Value
University of Witwatersrand	1	University of Johannesburg	107	University of Witwatersrand	1,376
		Council for Scientific and Industrial Research	95	University of KwaZulu Natal	1,165
		Rhodes University	59	University of Cape Town	693
		University of Pretoria	36	North West University	580
		North West University	30	NIPER	576
		Indian Institute of Science	17	University of Western Cape	518
		University of Western Cape	12	Council for Scientific and Industrial Research	377

### Funding instruments

**FIGURE 7:**
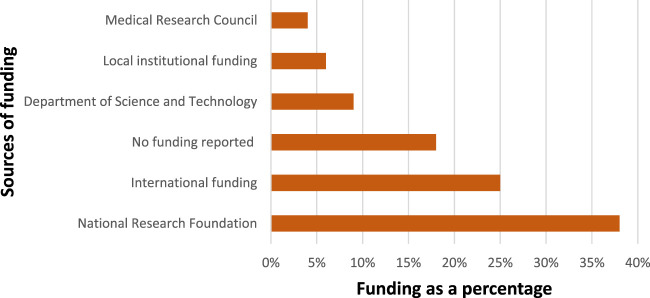
Source of funding of the scientific research in nanomedicine drug delivery.

Nanomedicine is a capital and resource intensive technology in which the journey from the laboratory to the market is long (Morigi et al., 2012). Industry and governments have made considerable investments in nanomedicine as the technology demands state of art equipment such as electron microscopes and clean room facilities. The funding is channeled towards running clinical trials as well as investigating new nanomedicine drug formulations in human subjects to understand the biological, biochemical, and biophysical mechanisms of living tissues. Manipulating materials at nanoscale through characterizing the molecular components inside cells at a level of precision is challenging and requires funding for procurement of the needed resources (McGoron, 2020). South Africa is one of the African countries that has made extensive investments toward creating a critical mass of infrastructure, equipment, and human capital for research in the application of nanomedicine (Dube and Ebrahim, 2017). The results of funding sources from the articles shows different funding streams. Figure 7 summarises the funding bodies as a percentage of the total.

The bulk of the funding was from the National Research Foundation (NRF) of South Africa which funded 38% of the studies. The NRF have different forms of funding nanotechnology, most notably is the Nanotechnology Flagship Projects (NFP) Grants which is designed to promote research projects that demonstrate the benefits of nanoscience and nanotechnology and their impact on some of the challenges facing South Africa. The focus of the funding could be one of the drivers for the increased research on the disease burden of the country such as TB. A further aim of NRF funding is emphasising human capacity development and collaboration, which could also account for several partnerships which are being formed resulting in the development of networks as shown in Figure 6. A further funding instrument by the NRF, encouraging collaboration, is the National Nanotechnology Equipment Programme which aims to position South Africa as a player in the emerging areas of nanoscience and nanotechnology, aligned with the objectives set forth in the National Nanotechnology Strategy.

International funding from countries such as India, Germany, South Korea, Saudi Arabia, Spain, Canada, Greece, United States of America and Malaysia constituted a quarter of the studies. With South Africa having well equipped centres of excellence in nanotechnology, the country provides an attractive site for the study of nanomedicine, hence the high number of international partners. The collaboration was mainly with developing countries of which India was the most dominant. India emerged as the largest collaborating partner for South Africa in nanomedicine research; the collaboration between South Africa and India could be due to the two countries have the similar disease burden. As an example, in 2016, India and South Africa were among the seven countries which accounted for two-thirds of the total new TB cases in the world (Floyd, Glaziou, Zumla and Raviglione, 2018). An analysis of the projects that were funded internationally reveal that some studies were not specific to a particular disease but focused mainly on the general mechanisms of drug delivery.

Eighteen percent of the articles reported no funding. This is interesting because nanomedicine is a resource intensive research field. The acknowledgements sections of studies revealed that access to the equipment for manipulating nanoscale particles was one of the important drivers for success of the projects. Several papers acknowledge access to laboratories and materials needed for research. Some studies even acknowledged the samples which were donated and made it possible to conduct the research without funding. Several researchers were able to conduct studies in nanomedicine by being given access to the laboratories and provided with materials and technical support (Branham, Moyo and Govender, 2012; Cosialls et al., 2017; Parboosing et al., 2017; Elemike, Onwudiwe, Nundkumar, Singh and Iyekowa, 2019). These interactions of accessing infrastructure and providing technical support could have culminated into new forms of collaborations.

The Department of Science and Innovation (DSI) had several joint funded projects with the NRF, and in some cases, it was difficult to make distinctions on the two. However, the funding from the DSI that was channeled through South African Research Chairs Initiative (SarChi) for Medicinal Chemistry and Nanotechnology was distinct. This funding was closely tied to nanomedicine and this research group, housed at Rhodes University, was active in the nanomedicine landscape in terms of research output. This could be because the SarChi initiative is designed to expand the scientific research base of the country in areas that are relevant to national development and global competence of the knowledge economy (Department of Science and Technology, 2019).

The national organisations, particularly universities and research institutions, also funded projects on nanomedicine. In many cases, organisational funding is provided as an additional support by the hosting organisation to augment the financial resources from the DSI and NRF. There are few exceptions of projects that were solely funded by a local university, and there were also projects jointly funded by two national universities. An analysis of the local organisations reveals that organisations that provided funding are among the most active actors involved in the exploration of nanotechnology in the country.

The South African Medical Research Council (SAMRC) funded significantly less studies than other local funders. The SAMRC have a mandate to promote the improvement of the health and quality of life of South Africans, and provides funding for basic laboratory investigations, clinical research, and public health studies. It focuses particularly on the South African burden of disease such as TB, HIV/AIDS, chronic diseases, alcohol and drug abuse, and women’s health. Although the SAMRC is regarded as the largest local funder of health research, it funded only four of these studies, and all four in collaboration with other funding sources.

## Discussion

Over the past 15 years, there has been a significant rise in the number of publications on nanomedicine drug delivery in South Africa, which is a positive indication of how South Africa is actively engaged with the technology. This, to some extent is an indication that the various measures been taken by the South African government in exploiting nanomedicine are paying off. An analysis of the results vis-à-vis the South African national nanotechnology strategy reveals that the country has made remarkable progress, especially in nurturing research on nanomedicine. The research as revealed by the publications of our search strategy have been mainly basic, which is important in developing the fundamental knowledge base upon which exploitative research can be done. Based on the findings from this study, strategic planning and policy interventions can be incorporated for development of nanomedicine in South Africa.

### From explorative to exploitative research

We showed a rising the number of published journal articles over time, particularly between 2016 and 2020. These publications are in reputable journals with about 73% of the lead authors being affiliated with South African organisations. This suggests that the national nanotechnology strategy has been instrumental in facilitating government efforts to build excellence in research and development capacity. The knowledge base for manipulating materials at a nanoscale has been developed. The 15-year period has allowed the researchers to be engaged in explorative research by searching, experimenting with, and developing new knowledge. What could be crucial in the coming years is for the researchers to engage more in exploitative research using the promising results from laboratory work. Although explorative and exploitative research may appear as contradictory activities, they can coexist and facilitate translating research findings into applications. The process of translating research into applications is regarded as the most challenging part which needs to be well orchestrated to ensure productive interactions. This should be strategically done so that the knowledge generated by different researchers can be exchanged while paying particular attention to the specific context of each country with the aim of promoting socially relevant research in healthcare. It may require creating a platform where researchers can interact with each other and share their expertise and challenges in the exploitation of nanomedicine.

### Enhancing collaboration and building networks

We have shown that strong networks have been established at national and international level. This is a goal of the national nanotechnology strategy which stipulates the need for the country to establish networking and shared resources. Interestingly, the collaborations are between both developing and developed countries, although the latter is still limited. An outlook of the networks indicates that while different universities and research institutions tend to interact with each other, some of the interactions are not direct but done through intermediaries. However, when it comes to exploitative research, it is not the collaboration in general that matters, but rather, its strength. Having strong networks is fundamental to generating scientific results with an impact on society as it entails higher trust and reciprocity, which has the effect of reducing the costs incurred and potential risks. Strong networks can induce higher propensity to produce scientific output of high impact. Thus, it is imperative that the networks are analysed in terms of their structure as the most influential partner(s) tend to be the ones located in central places.

The centrality metrics have been useful in determining the important actors in the network. When it comes to exploitative research, there are many issues at stakes, such as proprietorship of licenses or patents generated from research, hence there is a need for carefully selecting the potential collaborators. South African organisations are encouraged to protect their intellectual property derived from publicly financed research, under the Intellectual Property Rights from Publicly Financed Research Act, Act 51 of 2008. All South African publicly financed Universities and Research Institutions have a Technology Transfer Office, who is responsible for creating an entrepreneurial culture in the organisation. Unlike explorative research, where partnerships can be forged for a short period of time, exploitative research is often a long and winding process that requires strategic and sustainable alignment of collaborators who can contribute meaningfully towards the research. While international collaborations are important, national collaborations need to be promoted as well.

### Targeting diseases affecting the poor

Our analysis of the application of nanomedicine drug delivery in targeting various diseases show that they relate to health challenges affecting developing nations. The major targets are infectious and non-communicable diseases. There is a mix of infectious diseases such as TB and HIV/AIDS, and non-communicable diseases like cancer, the morbidity and mortality of which disproportionately affect people living in sub-Saharan Africa (Wong et al., 2021). For example, the region has the largest number of people living with HIV/AIDS in the world, while non-communicable diseases such as cancer are emerging as a threat, with a 20% possibility of one person dying from the ailments which is one of the highest in the world (Mendis, 2014). This implies that nanomedicine is being applied in diseases which are relevant to the African context. This is important for mobilising funding for research as there is a direct connection between what is being researched in the laboratories and its application in society. With the burden of diseases, particularly infectious diseases, being a major setback in the economic development of the country and continent, it is important to streamline research by prioritising specific diseases. This can be achieved through funding streams dedicated to the application of nanomedicine to particular diseases which are more prevalent in developing countries.

### Dedicating funding for nanomedicine

There are different sources of funding for research on nanomedicine. The South African government provides the bulk of the funding through the DSI and NRF. The funding is not directed to nanomedicine in particular or nanotechnology in general, except for the SarChi on Medicinal Chemistry and Nanotechnology hosted by Rhodes University and Pharmaceutical Biomaterials and Polymer-Engineered Drug Delivery Technologies, which is based at University of the Witwatersrand. The funding offered through SarChi is extensive, with an investment trajectory of up to 15 years. It is evident in the volumes of publications that came from the projects under that ambit. The other funding streams are not dedicated to nanomedicine, but are meant to cover science, technology, and innovation in its broad context. To stimulate innovation in nanomedicine, there is a need for incubation and technology transfer. To facilitate that, it is imperative that the South African government establishes dedicated funding for supporting the translation of nanomedicine research into products that benefit society. As the applications of nanotechnology are broad, is imperative to allocate financial resources for the sole purpose of providing a stable and reliable funding source for nanomedicine. Unlike other applications of nanoscience and nanotechnology, nanomedicine is unique in that there are complexities in the optimization of the formulation processes and achieving reproducibility. The uniqueness of nanomedicine calls for innovative approaches that entice researchers to invest their time and effort, thus the role of direct funding cannot be overemphasized.

### Moving towards the development of a regulatory framework

Nanomedicine presents complexities with the regulatory framework as the manipulation of materials at nanoscale induces risks and uncertainties that are not addressed by traditional regulatory approaches. As South Africa progress towards exploiting nanomedicine drug delivery, it is necessary to enact regulations that guide clinical trial phases prior to entry in the market and consider variables such as toxicity, solubility, bioavailability, and efficacy. Early engagement with legislation governing the use of nanomedicine is crucial so that measures can be put in place before the technology is commonplace. This is particularly important with poverty related diseases such as TB and HIV. There are significant regulatory factors which must be considered such as the toxicology and safety assessment due to risk profile of the materials at nanoscale. The recently enacted Medicines and Related Substances Amendment Act 14 of 2015 does not provide guidelines on the risk governance of nanomedicine. Thus, it is important that as nanomedicine is being developed for application, concerted effort should be made towards the enactment of regulations that emphasize all aspects related to safety testing and risk identification. Taking a pro-active approach towards developing a regulatory framework is crucial compared to being reactionary.

## Conclusion

The study has reviewed the evolution of the nanomedicine landscape in South Africa. This study reveals that substantial progress has been made in scientific knowledge development and in acquiring infrastructure for harnessing nanomedicine. There are several funding streams for nanomedicine, and the past 15 years have seen the development of a critical mass of skilled researchers across national universities and research institutions. Scientific publications have increased rapidly, while collaborations both nationally and internationally have been forged. These developments provide a strong base for the exploitation of nanomedicine to improve drug delivery in South Africa. However, there is need for co-ordination among funding bodies and researchers so that the country can focus on targeted applications of nanomedicine. Since nanomedicine may be broad and fragmented, it is imperative that the country occupy a particular niche and develop specific competence to move the technology from the lab to bench. This demands orchestrating research which can be instrumental in further exploitation of the technology.

## Appendix A

**Table:**

Intervention		
#1	Terms	Drug delivery OR Targeted drug OR Drug targeting
Technology		
#2	Terms	Nanotechnology OR Nanostructures OR Nanomedicine OR Nanotechnology
Geographical location		
#3	Terms	South Africa OR South African
#4	Combine #1 AND #2 AND #3	
#5	Filter by specifying the period	January 2006 to December 2020
